# Intestinal loads of extended-spectrum beta-lactamase and Carbapenemase genes in critically ill pediatric patients

**DOI:** 10.3389/fcimb.2023.1180714

**Published:** 2023-05-02

**Authors:** Elias Dahdouh, Emilio Cendejas-Bueno, Guillermo Ruiz-Carrascoso, Cristina Schüffelmann, Fernando Lázaro-Perona, Mercedes Castro-Martínez, Francisco Moreno-Ramos, Luis Escosa-García, Marina Alguacil-Guillén, Jesús Mingorance

**Affiliations:** ^1^ Clinical Microbiology and Parasitology Department, Hospital Universitario La Paz, Instituto de Investigación Sanitaria del Hospital Universitario La Paz (IdiPAZ), Madrid, Spain; ^2^ Centro de Investigación Biomédica en Red de Enfermedades Infecciosas (CIBERINFEC), Instituto de Salud Carlos III, Madrid, Spain; ^3^ Pediatric Intensive Care Unit, Hospital Universitario La Paz, Madrid, Spain; ^4^ Preventive Medicine Department, Hospital Universitario La Paz, Madrid, Spain; ^5^ Department of Pharmacy, Hospital Universitario La Paz, Madrid, Spain; ^6^ Pediatric Tropical and Infectious Diseases Department, Hospital Universitario La Paz, Madrid, Spain

**Keywords:** intestinal dominance, pediatric patients, β-lactamase genes, relative intestinal load, qPCR, antibiotic consumption, extra-intestinal multi-drug resistant organisms, microbiome

## Abstract

**Introduction:**

Intestinal colonization by Multi-Drug Resistant Organisms (MDROs) can pose a threat on the health of critically ill patients. The extent of colonization by these organisms is related to previous antibiotic treatments and their ability to cause infections among adult patients. The aim of this study is to determine the relationship between the intestinal Relative Loads (RLs) of selected antibiotic resistance genes, antibiotic consumption and extra-intestinal spread among critically ill pediatric patients.

**Methods:**

RLs of *bla*_CTX-M-1-Family_, *bla*_OXA-1_, *bla*_OXA-48_ and *bla*_VIM_ were determined in 382 rectal swabs obtained from 90 pediatric critically ill patients using qPCRs. The RLs were compared to the patients’ demographics, antibiotic consumption, and detection of MDROs from extra-intestinal sites. 16SrDNA metagenomic sequencing was performed for 40 samples and clonality analyses were done for representative isolates.

**Results and discussion:**

76 (74.45%) patients from which 340 (89.01%) rectal swabs were collected had at least one swab that was positive for one of the tested genes. Routine cultures did not identify carbapenemases in 32 (45.1%) and 78 (58.2%) swabs that were positive by PCR for *bla*_OXA-48_ and blaVIM, respectively. RLs of above 6.5% were associated with extra-intestinal spread of blaOXA-48-harboring MDROs. Consumption of carbapenems, non-carbapenem β-lactams, and glycopeptides were statistically associated with testing negative for *bla*_CTX-M-1-Family_ and *bla*_OXA-1_ while the consumption of trimethoprim/sulfamethoxazole and aminoglycosides was associated with testing negative for blaOXA-48 (P<0.05). In conclusion, targeted qPCRs can be used to determine the extent of intestinal dominance by antibiotic resistant opportunistic pathogens and their potential to cause extra-intestinal infections among a critically ill pediatric population.

## 1 Introduction

Intestinal colonization with Multi-Drug Resistant Organisms (MDROs) capable of producing Extended-Spectrum β-Lactamases (ESBLs) and/or carbapenemases is a global concern since these organisms can cause life-threatening opportunistic infections (Weinstein, 2012). Intestinal colonization by MDROs is often a result of antibiotic consumption that selects for these organisms and allows them to overgrow and dominate the intestinal microbiome (Willmann et al., 2019). This dominance poses an additional threat since it is linked to higher rates of infections (Sun et al., 2021) and transmission of MDROs (Lerner et al., 2015). Moreover, the resulting dysbiosis in the gut microbiome could have detrimental effects on human health (Young, 2017), and especially on the healthy development of children (Gaufin et al., 2018).

Recent studies have shown that the degree of intestinal colonization by opportunistic pathogens, especially ESBL- and carbapenemase-producers, can have a direct effect on infection and mortality rates (Dahdouh et al., 2021; Migliorini et al., 2022; Pérez-Nadales et al., 2022). Moreover, this colonization can be occult, *i.e.* present at such low amounts that it is not detected by traditional culture media, but has the potential to quickly expand and dominate the intestinal microbiome in response to antibiotic pressure (Sim et al., 2022). Previous studies have shown associations between high intestinal Relative Loads (RLs) of carbapenemase-producing *Klebsiella pneumoniae*, antibiotic consumption, and development of infections in hospitalized adult patient populations (Migliorini et al., 2022;, Lázaro-Perona et al., 2020), and in pediatric liver transplant patients (Dahdouh et al., 2022). The aim of this study is to use the qPCR assay developed and validated in these previous studies to determine the relationship between the intestinal RLs of selected ESBL and carbapenemase genes, antibiotic consumption, occult colonization, gut microbiome dysbiosis, and extra-intestinal spread of MDROs among critically ill pediatric patients.

## 2 Materials and methods

### 2.1 Study design

The study was retrospective and performed at the Hospital Universitario La Paz (HULP) in Madrid, Spain; a 1,200 bed tertiary care center. The pediatric patients included in this study were all those that had at least one stay at the pediatric Intensive Care Unit (pICU) of the hospital between April, 2018 and December, 2019. These patients were then followed throughout the duration of the study even when they were transferred, admitted, or visited other hospital wards at HULP. The samples received from these patients were originally collected for routine epidemiological surveillance and there wasn’t any sample that was collected specifically for this study. All the samples were re-used after fulfilling their diagnostic purposes and were analysed in bulk. Also, no results were communicated in real time to neither the clinicians nor the patients, and therefore the results obtained from this study did not interfere with the clinical management of the patients involved. Pediatric liver transplant patients were excluded since they received extensively more antibiotics than other patient groups and were included in a previous study performed by our group (Dahdouh et al., 2022).

During the time of the study, rectal swabs were routinely collected and sent to the microbiology department of HULP to determine the presence of ESBL, OXA-48, and VIM producers was implemented due to an ongoing outbreak caused by carbapenem-resistant *K. pneumoniae* that started in 2010 and has become endemic at the hospital (Pérez-Blanco et al., 2018). The rectal swabs were received from patients whose health condition allow for taking the swab and were initially cultured on MacConkey plates containing 4µg/mL cefotaxime (custom made by Maim^®^, Madrid, Spain) for the detection of ESBL producers, and on ChromID CARBA SMART™ agar plates (Biomérieux^®^, Marcy l’étoile, France) for the detection of carbapenemase producers. This was part of the routine epidemiological surveillance performed at HULP. After reaching the clinical conclusions, the same swabs were then collected to be reused in this study. Their contents were suspended in 0.5 mL of TE buffer (10mM Tris and 1mM EDTA; pH = 8.0) through vigorous shaking. The suspension was then stored at -20°C until used, and analysed in batches when a sufficient number of samples was reached. In the cases where the isolates obtained from the rectal swabs were stored in the hospital’s bacterial collection, they were recovered. Moreover, all the MDRO isolates that were obtained from clinical samples of the patients included in this study (such as urine, blood, abscesses, etc…), and that were stored in the bacterial collection as part of HULP’s routine practice were recovered to be used in this study. These latter isolates are labelled “extra-intestinal isolates” throughout the rest of the article.

### 2.2 DNA extraction

100µL of the rectal swab suspensions were diluted with 900µL of TE buffer in order to avoid inhibition of the qPCR reactions. Total DNA was then extracted by heating this suspension at 95°C for 20 minutes, performing mechanical lysis at 7,000rpm for 70 seconds using MagNA Lyser Green Beads and MagNa Lyser™ (Roche^®^, Mannheim, Germany), and extracted using the MagNA Pure Compact Nucleic Acid Isolation Kit I and MagNA Pure Compact™ system (Roche^®^, Mannheim, Germany). The DNA was then stored at -20°C.

### 2.3 Quantification of the relative intestinal loads of bla_CTX-M-1-Family_, bla_OXA-1_, bla_OXA-48_, and *bla*_VIM_ genes

The qPCR assays designed in one of our previous studies were used to determine the Relative intestinal Loads (RLs) of the beta-lactamase genes that are endemic at HULP (*bla*_CTX-M-1-Family_, *bla*_OXA-1_, *bla*_OXA-48_, and *bla*_VIM_) (Dahdouh et al., 2022). Briefly, reaction mixtures containing 10µl 2X PowerUP™ SYBR^®^ Green Master Mix (Applied Biosystems, Waltham, MA, USA), 0.05µM of each respective primer pair, 6µl H_2_O and 2µl DNA were prepared. The CFX Connect™ Real-Time System (BioRad, Madrid, Spain) was used for thermal cycling under the following conditions: 95°C for 3 minutes, then 40 cycles of 95°C for 15 seconds and 60°C for 1 minute; followed by melting curve analyses.

RLs of the tested genes were determined in relation to the C_t_ values of the *16SrDNA* gene and normalized to the RLs of these genes in pure bacterial cultures using the 2^-ΔΔCt^ method (Livak and Schmittgen, 2001). The RLs were then transformed into an inverse logarithmic scale where 0 represents RLs that are equivalent to pure bacterial cultures, –1 represents 10% of the bacterial population collected in the rectal swab, –2 represents 1%, and so on until –6 which represents 0.0001% of the total bacterial population, and our detection limit. The values are also expressed (where relevant) as percentages (%RL) where the values were converted to percentages using the formula 10^RL^ × 100.

### 2.4 Clonality analyses

Clonality analyses were done using Random Amplified Polymorphic DNA (RAPD) (Hsueh et al., 1998). The primers used were OPA-2 (5’-TGCCGAGCTG-3’), OPA-12 (5’-TCGGCGATAG-3’), and OPA-18 (5’-AGGTGACCGT-3’) and the thermal cycling conditions were 94°C for 5 minutes, followed by 45 cycles of 94°C for 1 minute, 37°C for 1 minute, and 72°C for 2 minutes, and a final step at 72°C for 2 minutes. PCR products were then visualized on 2% agarose gels and the resulting profiles obtained were used to determine the clonal relatedness of the isolates.

### 2.5 Next-generation sequencing

Whole-genomes were sequenced for representative isolates from the most frequent clones (as determined by RAPD). The isolates were cultured overnight on blood agar plates at 37°C and DNA was extracted from 10 single colonies as described previously (section 2.2). The NEBNext^®^ Fast DNA Fragmentation & Library Prep Set for Ion Torrent™ (New England Biolabs, Ipswich MA, USA) was used to prepare the libraries according to the manufacturer’s instructions. Mag-Bind^®^ TotalPure NGS beads (Omega Bio-Tek, Norcross GA, USA) was used for the purifications step, and the Ion Chef^®^ and S5^®^ Gene Studio (ThermoFisher Scientic, Waltham MA, USA) were used for sequencing. The reads were assembled using Geneious (v.10.1.3, Biomatter Ltd., Auckland, New Zealand). They were also tested for the presence of antibiotic resistance determinants and plasmids, and were allocated a specific Sequence Type (ST) using the tools available on the Center for Genomic Epidemiology website (https://cge.cbs.dtu.dk ).

*16SrDNA* metagenomic sequencing was performed to rectal swab suspensions that had varying RLs towards the tested genes, and the samples were labelled S1 through S40. Samples S1 to S29 were positive to at least one of the genes tested for, and were ordered in decreasing values of RLs while samples S30 to S40 were negative to all 4 tested genes. The Ion 16S™ Metagenomics Kit and the Ion Plus™ Fragment Library Kit with the Ion Xpress Barcode Adapters (Thermo Fisher, USA) kits were used to prepare the libraries according to the manufacturer’s instructions. Sequencing was performed using the same instruments as above and the sequences were analyzed using ION Reporter™ (Thermo Fisher, USA). All the sequences obtained from this study were deposited in Genbank under the Bioproject with the accession number PRJNA911601.

### 2.6 Patient data

Patient data (age, gender, colonization status, extra-intestinal infections 14 days before or after rectal swab collection, colonisation status upon sample collection, and antibiotics received) were retrieved from the hospital’s information system database. The patients were given study-specific codes in such a way that no patient can be identified. Approval of HULP’s local ethics committee was obtained for the study (PI-3428).

### 2.7 Statistical analyses

Normality of the data was tested for using the Kolmogorov-Smirnov (if n>50) and Shapiro-Wilk (if n<50) tests. The RLs of the four tested genes were grouped based on whether or not they correspond to an extra-intestinal infection, whether or not the patients received antibiotics in the 30 days prior to sample collection, and whether or not they correspond to detection of MDROs using the routine screening cultures performed at HULP. Two-sided student T-tests and ANOVA were used for normally distributed quantitative data, and Kruskal-Wallis and Mann-Whitney U tests were used for non-normally distributed quantitative data. Chi-squared was used for the comparison of categorical variables such as antibiotic consumption in relation to having one or more β-lactamase genes detected in the rectal swab. Receiver Operating Characteristic (ROC) analyses were performed using the easyROC online tool (http://biosoft.erciyes.edu.tr/app/easyROC/) in order to determine cutoff values after which there is an increase rate of detection of extra-intestinal MDROs harbouring the same gene as that detected in the rectal swabs. The Youden method (*i.e.* determining the J value through the formula [sensitivity + specificity – 1] for all the points of the curve) was used in order to determine the cutoff values. Statistical significance was determined when *p* values were less than 0.05 and all the statistical tests were performed using SPSS (version 24.0; IBM, Armonk, NY, USA).

## 3 Results

### 3.1 Clinical data and detection of β-lactamase genes

The general characteristics of the patients and the samples included, as well as the qualitative results obtained by routine cultures and qPCRs are presented in **Table 1**. Of the 90 patients included in this study, 42 were chronic. Of these chronic patients, 19 were transplant patients (9 kidney, 4 multi-organ, 3 hematopoietic, and 3 cardiac transplants). Of the 382 rectal swabs obtained from the 90 patients included in the study, 317 (82.98%) were collected from chronic patients and 65 (17.02%) were collected from acute patients (2 or less swabs from each acute patient that had average hospital stays of less than 1 week). Sixty-seven patients from which 340 rectal swabs were collected had at least one swab that was positive for at least one of the genes tested by PCR. Thirty-eight of these patients had both ESBLs and carbapenemases, 18 had only carbapenemases, and 11 had only ESBLs. Just 3 of the 23 patients that were negative for these genes where chronic patients (15 (4.73%) samples), and the rest were acute patients (40 (61.54%) samples). By routine cultures, 28 patients had at least one sample positive for ESBLs, 24 for carbapenems, and 10 for both carbapenems and ESBLs.

**Table 1:** Description of the patients and samples included in this study, as well as the qualitative results obtained by routine cultures and qPCRs for the genes included in this study.

General Characteristics of the Patients and Samples Included in the Study	
**Number of Patients**	90
**Chronic Patients^1^**	42
**Acute Patients^2^**	48
**Average Age (years)**	8.3 (median = 6; range = 3 – 18)
**Gender**	43 (48.8%) Female
**Average of Weeks Followed**	11 (median = 6; range = 0.3 – 57)
**Average number of Swabs per Patient**	4 (range: 1 – 23)
**Total Rectal Swabs Collected**	382
**Paediatric Intensive Care Unit**	130 (34%)
**Haematology/Oncology Outpatient**	54 (14.1%)
**Emergency**	40 (10.5%)
**Haematology/Oncology Inpatient**	39 (10.2%)
**Paediatrics**	36 (9.4%)
**Nephrology Inpatient**	25 (6.5%)
**Gastroenterology**	22 (5.8%)
**Neonatal**	11 (2.9%)
**Nephrology Outpatient**	10 (2.6%)
**Surgery**	8 (2.1%)
**Cardiology**	7 (1.8%)
**Routine Epidemiological Screening**	
**Carbapenemase-Producers**	82 (21.5%)
**ESBL-Producers**	53 (13.9%)
**ESBL- and Carbapenemase-Producers**	9 (2.4%)
**Negative**	211 (55.2%)
**Not Processed**	27 (7.1%)^3^
ESBL Producers Detected by Routine Screening (n = 55)^4,5^	
***Klebsiella pneumoniae***	45 (81.8%)
***Enterobacter cloacae***	4 (7.3%)
**Others (incidence of 3 or less)**	6 (10.9%)
Carbapenemase Producers Detected by Routine Screening (n = 83)^4,5^	
***Klebsiella pneumoniae***	50 (60.2%)
***Enterobacter cloacae***	11 (13.3%)
***Klebsiella oxytoca***	7 (8.4%)
***Escherichia coli***	9 (10.8%)
**Others (incidence of 3 or less)**	6 (7.2%)
Genes Detected by PCR^6^	
***bla*_CTX-M-1-Family_**	136 (35.6%)
***bla*_OXA-1_**	109 (28.5%)
***bla*_OXA-48_**	71 (18.6%)
***bla*_VIM_**	134 (35.1%)
**Negative for all 4 genes**	122 (31.9%)

^1^Chronic patients included pediatric transplant (excluding liver transplant) and haematological/oncological patients.

^2^Acute patients included accident victims and neonates with post-partum complications. ^3^These rectal swabs were not processed by routine culture screening because they did not comply with the standard protocols applied at the Hospital Universitario La Paz at time of the study.

^4^There were multiple instances of more than one organism being isolated from a single rectal swab.

^5^8 Klebsiella pneumoniae isolates and 1 K. oxytoca isolate had both ESBL and Carbapenemases.

^6^There were multiple instances of more than one gene detected in the same rectal swab.

*K. pneumoniae* was the most frequent species isolated from the rectal swabs (**Table 1**). To study the relationship between the different isolates, clonality analyses through RAPD was performed for 43 isolates that could be recovered from 21 patients. The most frequent clone comprised 20 isolates originating from 9 different patients, and was found (through whole-genome sequencing) to belong to sequence type (ST) ST39. Another clone was found to belong to ST37 and was detected in 7 isolates from a single patient. A third clone was detected in 2 isolates obtained from a single patient and belonged to ST3155, and a fourth clone that belonged to ST1928 was also detected in 2 isolates obtained from another patient. The rest of the isolates (twelve isolates from nine patients) included in the clonality analyses did not have a profile that was similar to any other isolate.

In total (out of the 382 rectal swabs), 136 (35.6%) were positive for *bla*_CTX-M-1-Family_, 109 (28.5%) for *bla*_OXA-1_, 71 (18.6%) for *bla*_OXA-48_, and 134 (35.1%) for *bla*_VIM_ by PCR. Compared to the PCR, the sensitivity of the routine cultures for the detection of OXA-48 producers was 51.52% and its specificity 98.39%. For VIM producers, the sensitivity was 38.1% and the specificity 98.98% (**Supplementary Table 1**). ESBL-producers were not included in this analysis since ESBL-harboring *E. coli* were reported as negative in the hospital’s information system database, according to the activated protocols at the time of the study.

### 3.2 Relative intestinal loads of the β-lactamase genes

**Figure 1:**
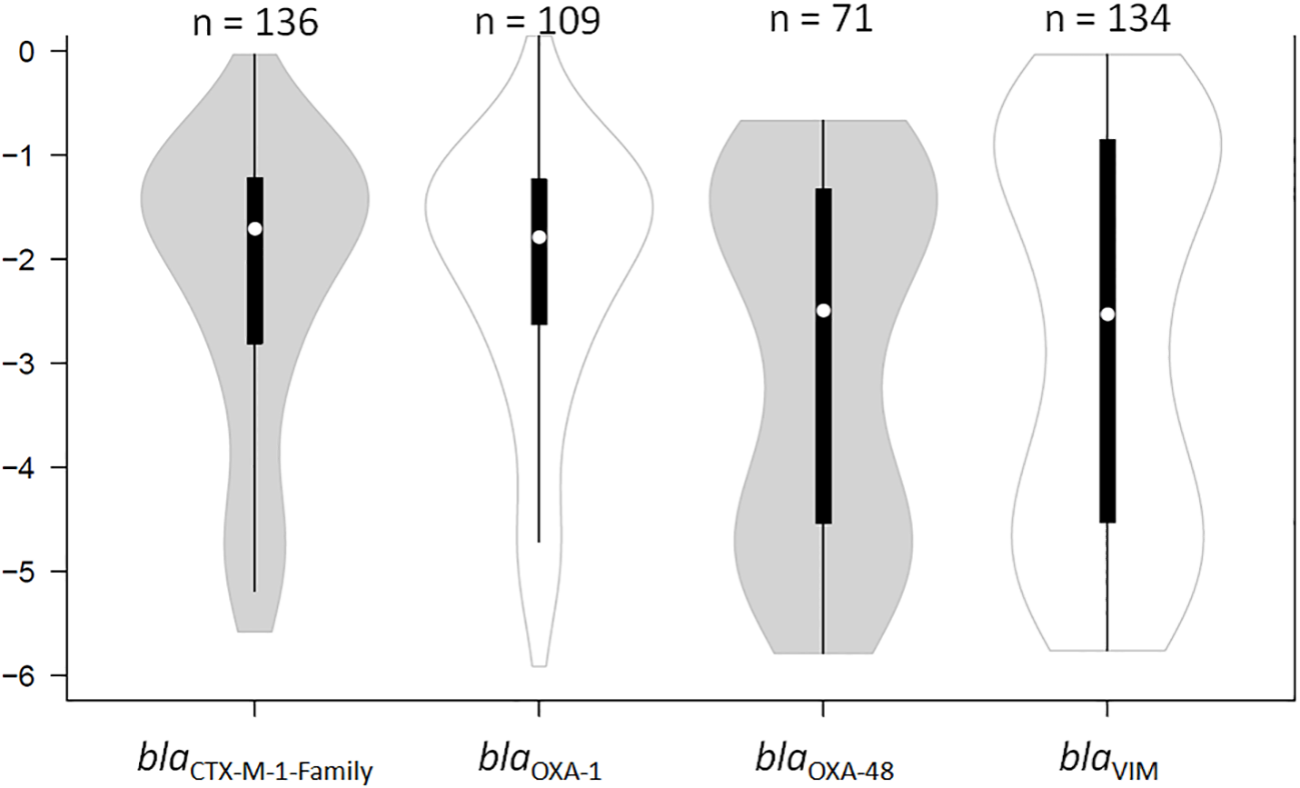
Violin plot showing the distribution of the relative loads of the samples that were positive by qPCR for *bla*_CTX-M-1-Family_, *bla*_OXA-1_, *bla*_OXA-48_, and *bla*_VIM_. The results are expressed in an inverse logarithmic scale where 0 is 100% of the bacterial population, -1 is 10%, -2 is 1%, and so on until -6 which is 0.0001% of the bacterial population, and our detection limit. “n” stands for the number of samples that were positive for the respective gene of antibiotic resistance.

The RLs of the four β-lactamase genes endemic at HULP (*bla*_CTX-M-1-Family_, *bla*_OXA-1_, *bla*_OXA-48_, and *bla*_VIM_) were determined through normalizing the Ct values of these genes to that of the *16SrDNA* gene. The RLs of the four genes measured in all the swabs fell within a six log range, from almost pure cultures (0; 100% of bacterial population) to -6 (0.0001% of the total bacterial population). The median RLs of *bla*_CTX-M-1-Family_ and *bla*_OXA-1_ were significantly higher than those of *bla*_OXA-48_ and *bla*_VIM_ (-1.7 (1.98%) and -1.79 (1.64%) versus -2.49 (0.32%) and -2.41 (0.4%), respectively; *P* < 0.05; **Figure 1**). The RLs determined for *bla*_OXA-48_ and *bla*_VIM_ were more evenly spread out across the detection ranges, but showed a bi-modal distribution pattern where they clustered in either high RLs (over -2 (*i.e.* 1% of the total bacterial population)) or low RLs (under -4 (*i.e.* 0.001% of the total bacterial population)) (**Figure 1**). Samples that were PCR-positive for *bla*_CTX-M-1-Family_ and *bla*_OXA-1_ often showed RLs that were higher than -2 (1% of the total bacterial population). There was no difference in RLs between male and female patients, nor was there any difference based on the age of the patient, the type of disease for which the patient was receiving treatment, the wards in which the patients were admitted to at the time of sampling, and whether or not the patients were critically ill.

**Figure 2:**
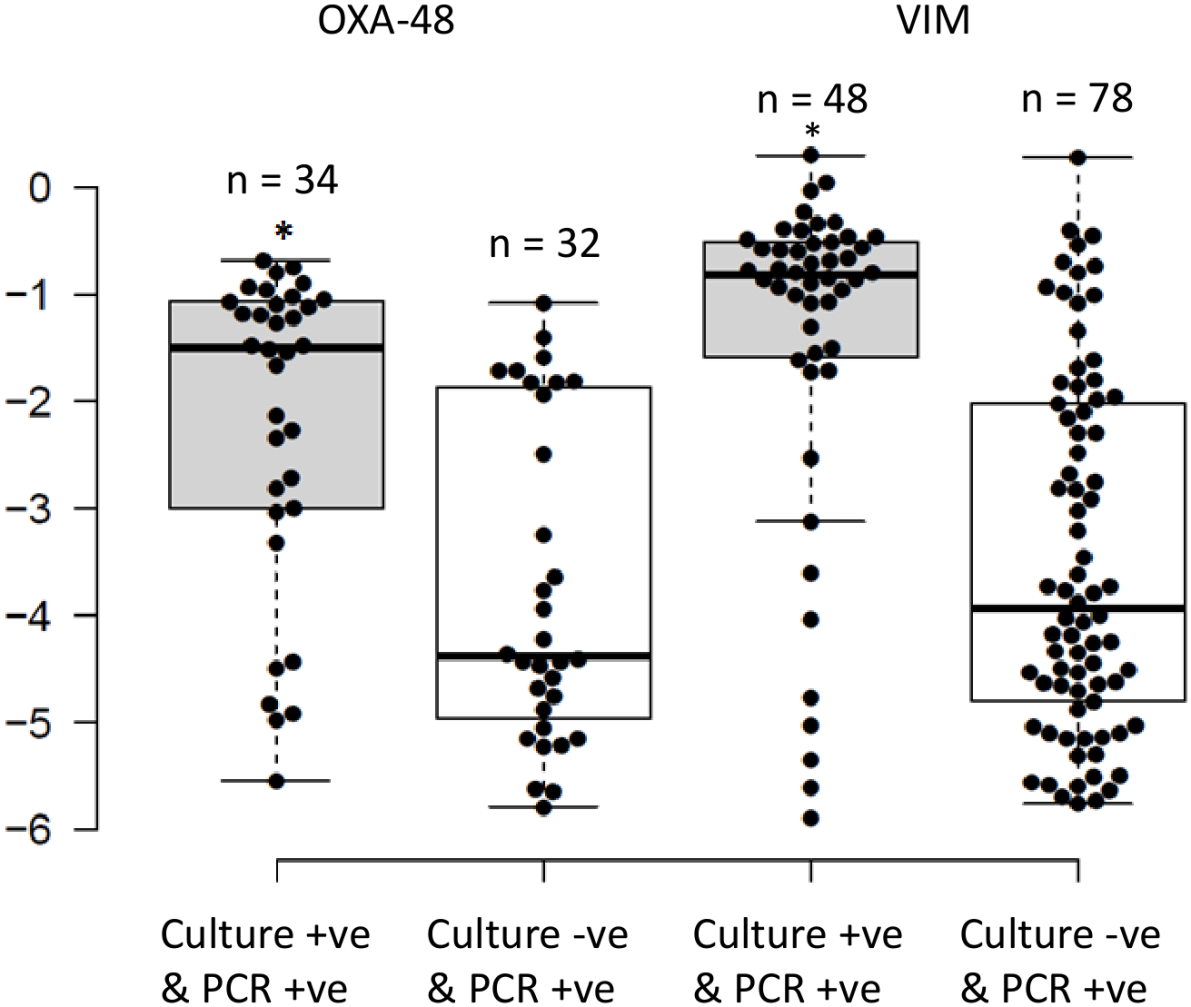
Distribution of the relative loads of the samples that were positive by routine cultures and PCR for OXA-48 and VIM, compared to those that were only positive by PCR. The results are expressed in an inverse logarithmic scale where 0 is 100% of the bacterial population, -1 is 10%, -2 is 1%, and so on until -6 which is 0.0001% of the bacterial population, and our detection limit. *Implies statistically higher relative intestinal loads for samples that were positive by PCR and routine cultures as opposed to being positive by PCR alone (*P*<0.05).

There were 75 (70.1%), 32 (45.1%), and 78 (58.2%) swabs positive by PCR for *bla*_CTX-M-1-Family_, *bla*_OXA-48_, and *bla*_VIM_ genes, respectively, that were negative in antibiotic-selective cultures. However, according to HULP’s protocols at the time of the study, ESBL-harboring *E. coli* were reported as negative in the hospital’s information system database, and therefore the total number of positive cultures for ESBL-producing organisms cannot be known. Comparing the RLs of the carbapenemase genes to the results of the routine cultures showed that the median RLs of *bla*_OXA-48_ and *bla*_VIM_ were significantly higher in samples that were positive by PCR and culture as compared to those only positive by PCR (-1.45 (3.2%) and -0.82 (15.1%) versus -4.38 (0.004%) and -4.02 (0.01%); respectively; *P*<0.05; **Figure 2**). Moreover, the bi-modal distribution pattern disappeared where the RLs of the samples that were positive through both methods clustered in the higher RL ranges while those that were only positive by PCR clustered in the lower RL ranges (**Figure 2**). The *bla*_OXA-1_ gene was not included in this analysis since it was not screened for in the routine cultures.

### 3.3 Antibiotic consumption and relative intestinal loads of antibiotic resistance genes

Antibiotic consumption data was retrieved from the hospital’s information system database for the patients that were included in this study throughout the duration of the study, and 30 days before inclusion. Twenty one (23.33%) patients did not receive any antibiotic throughout the study period, and in 37 (41.11%) patients, all the samples collected from them corresponded to them receiving antibiotics during the previous 30 days. These treatments were not consecutive and were often mixed where different antibiotics were received at different times, but they always received at least one type in the 30 days before sampling. The remaining 32 (35.56%) patients were mixed where some swabs corresponded with antibiotic consumption during the 30 days before sample collection, and others did not. From the first group of 21 patients, 42 (10.99%) rectal swabs were collected, and from the second group of 37 patients 139 (36.39%) swabs were collected. From the third group of mixed patients, 138 (36.12%) rectal swabs corresponded with antibiotic consumption and 63 (16.49%) of them did not correspond with the patient receiving antibiotics in the previous 30 days.

In total, 277 (72.51%) of the rectal swabs corresponded with antibiotic consumption during the 30 days prior to collection, and 105 (27.49%) did not. For 72 rectal swabs, the patients received one type of antibiotic, for 40 rectal swabs the patients received antibiotics from 2 different classes, and for the remaining 165 swabs the patients received 3 or more antibiotics from different classes. **Table 2** shows the type of antibiotics that the patients received before sample collection. In the majority of the incidents (82%), the antibiotics were administered intravenously, in 14.5% of the incidents antibiotics were received orally and intravenously, and in 3.5% of the incidents the antibiotics were only administered orally.

**Table 2:** Distribution of samples in light of antibiotic consumption in the 30 days prior to the collection of the rectal swab.

Antibiotic Consumption up to 30 Days Before Sample Collection		
Patients With no Antibiotics Received	21	
Patients that Received Antibiotics	69	
Samples Collected with no Antibiotics Received	105	
Samples Collected from Patients that Received Antibiotics	277	
**Number of Antibiotics Received (by Class)**	Number of Rectal Swabs	
**1**	72	
2	40	
3 or more	165	
**Antibiotics Received**	Number of Rectal Swabs	Median Days Received
Non-Carbapenem β-Lactams	217	3 (range: 1 – 38)
Glycopeptides	160	5 (range: 1 – 29)
Carbapenems	124	6 (range: 1 – 29)
Aminoglycosides	104	2 (range: 1 – 27)
Trimethoprim/Sulfamethoxazole	83	3 (range: 1 – 25)
**Others**	390	3 (range: 1 – 30)

^1^The other antibiotics received are macrolides, fluoroquinolones, linezolid, and the antifungals fluconazole, metronidazole, micafungin, and voriconazole.

The consumption of carbapenems, non-carbapenem β-lactams, and glycopeptides was significantly associated with testing negative for *bla*_CTX-M-1-Family_ and *bla*_OXA-1_ (*P* < 0.05). Consumption of aminoglycosides and Trimethoprim/Sulfamethoxazole (TMX) was also significantly associated with testing negative for *bla*_OXA-48_ (*P* < 0.05). Additionally, the consumption of TMX was significantly associated with lower median RL values for *bla*_OXA-48_ (-1.82 (1.5%) versus -4.45 (0.004%); *P* < 0.05). No statistically significant associations were determined for the rest of the antibiotic classes and the other antibiotic resistance genes.

### 3.4 Intestinal dominance by bacteria harboring β-lactamase genes and extra-intestinal spread

The RLs determined for the four genes tested for were compared to incidents where extra-intestinal isolates were collected within 14 days of rectal swab collection. Thirty-three extra-intestinal β-lactam resistant isolates have been registered in the hospital information system from 18 patients included in the study. Fourteen isolates were obtained during the two weeks before the collection of the rectal swab, seven on the same day, and twelve during the two weeks after the collection of the rectal swabs. These incidents were at least 2 weeks apart when collected from the same patient in order to exclude ongoing infections caused by the same isolate. Twenty of the extra-intestinal isolates were related to infections while the remaining thirteen were considered colonisations, as registered by the attending physician at the time (**Table 3**). In 28 of these episodes (84.85%), the patients received antibiotics up to 30 days before extra-intestinal detection of MDROs. It was possible to compare the extra-intestinal isolates to the intestinal ones in only 21 of these cases (13 of them were infections) since in the remaining cases, no growth was reported through routine culture from the corresponding rectal swab. In 16 of these 21 (76.2%) cases the same organism was detected in both the rectal swabs and the extra-intestinal site. Clonality analyses through RAPD comparing these isolates was only possible for 9 incidents (where the isolates were stored in the hospital’s bacterial collection), and in 8 of them (88.9%) the same clone was detected. In terms of the β-lactam resistance genes, in out 27 of the 33 isolates (81.8%), the same gene was detected in the intestinal (detected through qPCR) and extra-intestinal sites (registered in the hospital’s database).

**Table 3:** Extra-intestinal detection of isolates resistant to β-lactams.

Extra-Intestinal β-Lactam Resistant Isolates	
**Extra-intestinal β-lactam resistant isolates**	33
**Bacteraemias**	5
**Surgical wound infections**	3
**Ventillator-associated pneumonias**	3
**Conjuctivitis**	2
**Pneumonias**	2
**Pyelonephritis**	1
**Sepsis**	1
**Surgery-related peritonitis**	1
**Pericatheter skin infection**	1
**Traqueobronchitis**	1
**Colinisations**	13
**Asymptomatic bacteruria**	5
**Broncheal aspirate**	5
**Skin**	2
**Pharyngeal swab**	1

**Figure 3:**
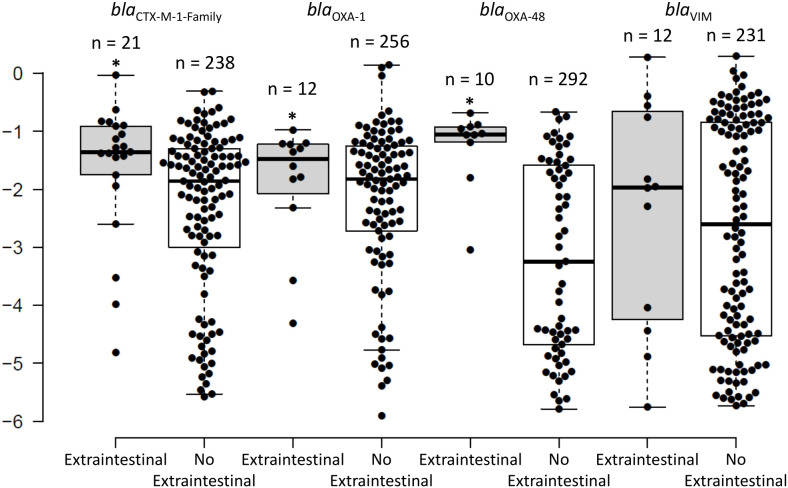
The distribution of the intestinal relative loads of the *bla*_CTX-M-1-Family_, *bla*_OXA-1_, *bla*_OXA-48_, and *bla*_VIM_ genes within 2 weeks of extra-intestinal isolation of an organism harbouring the same gene, as compared to the intestinal relative loads that do not correspond to extra-intestinal isolation of an organism harbouring these genes. * signifies statistically higher relative loads for the swabs corresponding with an extra-intestinal isolate compared to those that do not.

The intestinal RLs of the antibiotic resistance genes collected within 2 weeks (average = 2 days; median = same day) from the extra-intestinal episode had median RLs of -1.36 (4.4%) for *bla*_CTX-M-1-Family_, -1.48 (3.5%) for *bla*_OXA-1_, -1.05 (8.9%) for *bla*_OXA-48_, and -1.97 (1.1%) for *bla*_VIM_. The RLs of *bla*_CTX-M-1-Family_ and *bla*_OXA-48_ were significantly higher in the samples close to an episode of extra-intestinal spread than in samples that were not associated to extra-intestinal episodes (-1.36 (4.4%) and -1.05 (8.9%) versus -1.85 (1.4%) and -3.25 (0.06%), respectively; *P* < 0.05; **Figure 3**). No statistical significance was detected for *bla*_VIM_. There were 11 extra-intestinal samples that corresponded with reporting of a culture-negative rectal swab. Seven of these samples corresponded with infections (bacteremia, peritonitis, and lower respiratory tract infections), and in all of them except for one, the same gene was detected in the rectal swab through PCR compared to the gene reported in the extra-intestinal isolate. In the seventh sample, no antibiotic resistant gene was detected in the rectal swab and an ESBL-producing organism was reported to cause ventilator associated pneumonia. Also among these 11 samples, 7 corresponded with the patient receiving antibiotics and the rest did not correspond with antibiotic consumption during the 30 days before sample collection. In the 4 remaining episodes, nothing was detected by PCR in the rectal swabs and all 4 episodes were registered as asymptomatic bacteruria.

**Figure 4:**
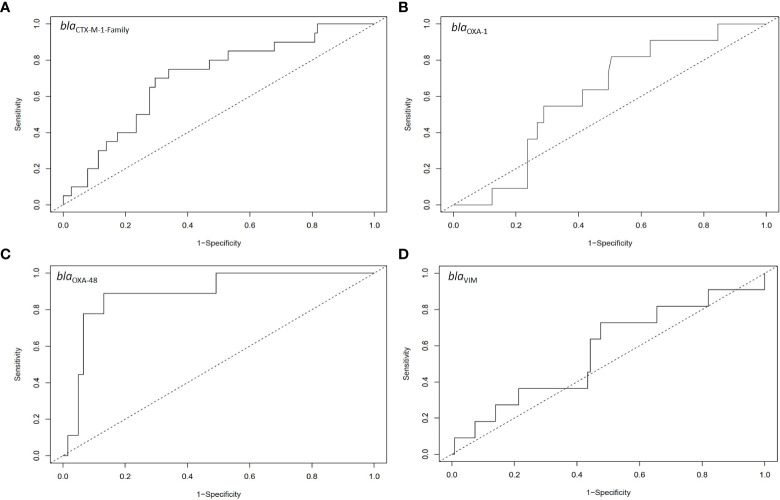
ROC analysis for the relative intestinal loads of **(A)** *bla*_CTX-M-1-Family_, **(B)** *bla*_OXA-1_, **(C)** *bla*_OXA-48_, and **(D)** *bla*_VIM_ for samples that had extra-intestinal isolates harbouring the relative genes as compared to those that did not have extra-intestinal isolates harbouring these genes. The number of samples included in these analyses are the same as those in **Figure 3**.

ROC analysis (Youden method) of the capacity of the RLs to identify samples close to an extra-intestinal spread episode determined a cut-off value of 6.5% for organisms harbouring *bla*_OXA-48_ (area under the curve = 0.89, sensitivity = 0.89, specificity = 0.87; *P* < 0.05; **Figure 4C**). In none of the other three genes the intestinal RL could identify samples close to extra-intestinal spread episodes (**Figure 4A, B, D**).

### 3.5 Microbiome diversity and relative intestinal loads

**Figure 5:**
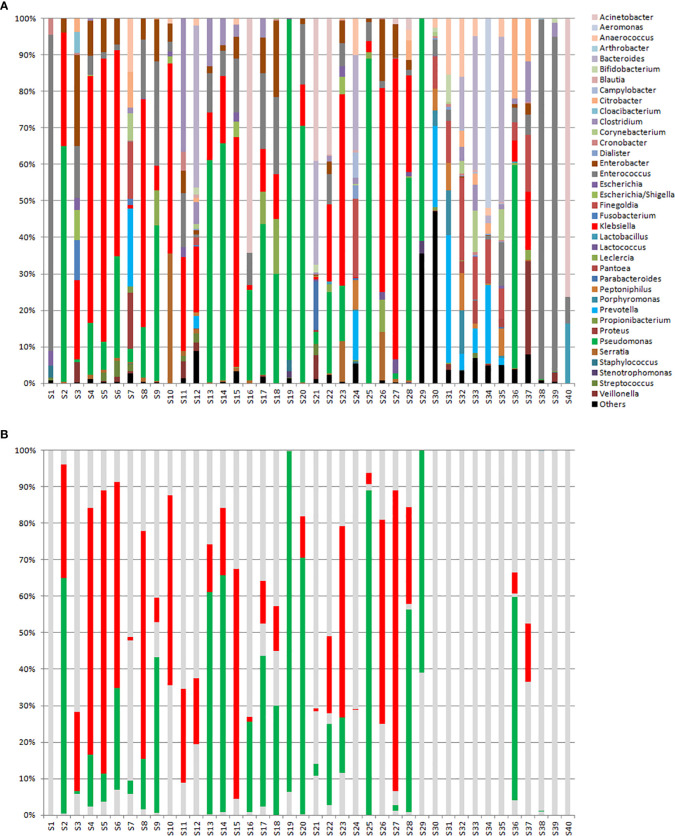
**(A)** *16sDNA* sequencing results for samples S1 to S29 that were ordered according to decreasing relative intestinal loads to one or several of the genes tested for, and samples S30 to S40 that were negative to all genes tested for. **(B)** The same samples shown in Part A but where only *Klebsiella* spp. (in red) and *Pseudomonas* spp. (in green) are highlighted.

Metagenomic analyses (*16SrDNA*) were done with the same rectal swab suspensions used for qPCR in a subset of forty selected samples (S1 to S40) (**Figure 5**). **Figure 5B** shows the same samples but where only *Klebsiella* spp. (red) and *Pseudomonas* spp. (green) are highlighted. These two organisms were chosen since, according to the local epidemiology at HULP, were the most likely organisms to harbour the genes tested for in our study. The observed number of genera, α-diversity (calculated according to the Shannon method), and percent relative loads (%RLs) of the four genes tested for in these samples are shown in **Supplementary Table 2**.

Comparing the positive samples to the negative ones, a higher diversity among the negative samples was observed (median of 31 genera versus 15 genera; *P*>0.05). Moreover, looking at the percentage of *Klebsiella* spp. and *Pseudomonas* spp. (**Figure 5B**), it is evident that all but 4 samples that were positive for at least one of the tested genes were dominated by one or both of these organisms, while in all but 2 samples that were negative for these genes these organisms were almost absent. Of the 4 positive samples that showed an exception, S1 was dominated by *Enterococcus* spp., S7 and S24 were relatively diverse, and S21 showed dominance by *Acinetobacter* spp. It is also interesting to note that the negative samples S38 and S39 were dominated by *Enterococcus* spp. and S40 was dominated by *Acinetobacter* spp. For samples S21 and S24, ESBL- and OXA-48-producing *K. pneumoniae* were registered in the hospital’s information system database, respectively. For samples S1, S38, S39, and S40, no record of a β-lactam resistant organism in the rectal swabs was registered in the hospital’s database system.

## 4 Discussion

In this study, we analyzed the intestinal RLs of the four genes of β-lactamases that are endemic at our hospital (*bla*_CTX-M-1-Family_, *bla*_OXA-1_, *bla*_OXA-48_, and *bla*_VIM_) using a previously validated qPCR assay (Dahdouh et al., 2022). The %RLs of *bla*_CTX-M-1-Family_ and *bla*_OXA-1_, which are commonly found on the same plasmid (Livermore et al., 2019), for the most part had values that ranged between 1% (-1) and 10% (-2) of the total bacterial population. However, those of *bla*_OXA-48_ and *bla*_VIM_ showed a bi-modal distribution where they were either very high or very low, with relatively few samples in between (**Figure 1**). This can have very important implications for intestinal colonizations with carbapenemase-producing organisms since they can be present at low RLs, which routine cultures are more prone to miss (**Figure 2**), but can quickly dominate the intestine and rise to high RLs with an increased probability of spreading into extra-intestinal sights and cause infections (**Figure 3**). Moreover, the bi-modal distribution could be related to the different pharmacokinetic/pharmacodynamic properties of antibiotics that affect these genes where they could be present in the gut in sub-inhibitory concentrations and allow these organisms to persist in low RLs (Sadgrove and Jones, 2019), a phenomenon that is not as observed for ESBL-producers. These latter organisms were, for the most part, colonizing the intestines at high RLs (**Figure 1**). This might be due to carbapenems, non-carbapenem β-lactams, and glycopeptides inhibiting the growth of ESBL carriers even at low amounts, as seen through the associations between testing negative for the ESBL genes and consumption of these antibiotics, making it unlikely that these organisms colonize at low RLs. This could also explain why they are almost constantly encountered at high RLs since these, or other antibiotics, can kill off the bacterial community and allow these ESBL producers to survive and dominate the intestines if they acquire additional mechanisms of antibiotic resistance that were not tested for in this study.

The data we obtained in this study from a heterogeneous pediatric population is somewhat different from that obtained for adults, where the %RLs of *bla*_OXA-48_ were mostly above 10% of the bacterial population (Lázaro-Perona et al., 2020). They were also different from those determined for pediatric liver transplant patients that receive prolonged and repeated treatments with antibiotics (Dahdouh et al., 2022). Nevertheless, in all three studies, the consumption of antibiotics was associated with intestinal RLs, where in adults the consumption of non-β-lactams and carbapenems were associated with high RLs for *bla*_OXA-48_ (Lázaro-Perona et al., 2020), and additionally to *bla*_CTX-M-1-Family_ and *bla*_OXA-1_ for pediatric liver transplanted patients (Dahdouh et al., 2022). In contrast, this current study showed that the consumption of β-lactams and glycopeptides is associated with a lower incidence of detecting *bla*_CTX-M-1-Family_ and *bla*_OXA-1_, and the consumption of aminoglycosides and TMX with a lower incidence of detecting *bla*_OXA-48_ in the critically ill pediatric patient population. The main reason for this difference between these studies is likely due to the highly heterogeneous nature of the patient population of this current study where different developmental stages, underlying conditions, co-morbidities, and treatments received with their specific pharmacokinetic/pharmacodynamic properties can make it difficult to obtain clear conclusions (Sadgrove and Jones, 2019; Ramirez et al., 2020; Shah et al., 2021). It might also be related to the short hospital stays for a significant number of included patients were the MDROs might not have had the chance to colonize their intestines. Nevertheless, this can have important implications for choosing the right antibiotics that can help in clearing an infection, as well as decrease the intestinal RL of MDROs. Further studies will be required to determine which antibiotics can achieve this optimum equilibrium between curing the patients and lowering the extent, or even eliminating, intestinal colonization by MDROs.

Despite the different effects that were observed in relation to antibiotic consumption, all three studies performed at our lab (including this one), and studies performed by other groups (Sun et al., 2021; Migliorini et al., 2022; Pérez-Nadales et al., 2022) demonstrated a relationship between high intestinal RLs and increased risk of extra-intestinal spread and subsequent infections by MDROs. Our study additionally shows intestinal dominance of the gut microbiome by a few organisms among the samples that were positive for antibiotic resistance genes in 26 out of 29 samples. Nevertheless, our method missed intestinal dominance in 5 samples that were negative for all the tested genes (**Figure 5**), most likely due to them harboring antibiotic resistance genes or mechanisms that were not tested for. This highlights another limitation of this current study and the need to develop more inclusive assays in case this approach is to be adapted to the routine practice. However, through further development and fine-tuning, our approach can prove to be valuable in being able to predict the effect of different treatments on intestinal dominance, make it possible to track this dominance over time, and take preventive measures that can stop the extra-intestinal dissemination of MDROs, especially since they are capable of causing infections. This is also interesting since, through this approach, very small RLs of MDROs (that are otherwise missed) can be detected and frequent sampling can trace a rise in RLs, allowing for clinical interventions before these organisms dominate the intestinal microbiome. Moreover, this approach can be combined with the local epidemiology at any hospital, making it adaptable and expandable for its applications where it can be directed towards the most common local genes of antibiotic resistance, opportunistic pathogens, and even dominant clones. Further studies will be directed at determining the clinical impact of this approach, especially in light of the patients’ clinical progression over time and in light of all the other clinical factors.

Taken together, our data suggests that using qPCR for the determination of the intestinal loads of resistance genes is more accurate than relying on routine cultures in critically ill pediatric patients. It also shows a link between lower frequencies of *bla*_CTX-M-1-Family_ and *bla*_OXA-1_ and the consumption of non-β-lactams and carbapenems, and lower frequencies of *bla*_OXA-48_ and the consumption of TMX and aminoglycosides. Finally, we showed a link between intestinal dominance determined by our assay, and extra-intestinal spread of MDROs, making this tool a valuable one that can be used to track the extent of colonization in critically ill pediatric patients.

## Data availability statement

All the data generated or analyzed during this study are included in this article and its supplementary material files. The obtained sequences were deposited in Genbank under the Bioproject with the accession number PRJNA911601. Further enquiries can be directed to the corresponding author.

## Ethics statement

The studies involving human participants were reviewed and approved by Comite Etico de Investigacion Clinica del Hospital Universitario La Paz (PI-3428). Written informed consent from the participants’ legal guardian/next of kin was not required to participate in this study in accordance with the national legislation and the institutional requirements.

## Author contributions

ED performed the lab work described in this article and drafted the manuscript. FM-R, EC-B, CS, and LE-G tracked the patients involved in the outbreak, collected the clinical data, and participated in acquiring the samples. GR-C identified the rectal swabs included in the study and performed the bacterial cultures. FL-P performed part of the whole-genome sequencing and clonality analyses. MC-M performed data analyses and recovered part of the data from the hospital’s information system database. MA-G performed part of the PCRs and clonality analyses. ED and JM designed the project. JM supervised the project. All authors contributed to the article and approved the submitted version.
