# Supplementary material for: Intestinal loads of extended-spectrum beta-lactamase and Carbapenemase genes in critically ill pediatric patients
Source: Front Cell Infect Microbiol. 2023 May 2;13:1180714. doi: 10.3389/fcimb.2023.1180714 (PMC10188119; doi:10.3389/fcimb.2023.1180714)
Supplement: Supplementary file 1 [file DataSheet_1.pdf]

Intestinal Loads of Extended-Spectrum Beta-Lactamase and Carbapenemase Genes in Critical Pediatric Patients

Elias Dahdouh[1*], Emilio Cendejas-Bueno[1,6], Guillermo Ruiz-Carrascoso[1,6], Cristina Schüffelmann[2], Fernando Lázaro-Perona[1], Mercedes Castro-Martínez[3], Francisco Moreno-Ramos[4], Luis Escosa-García[5,6], Marina Alguacil-Guillén[1], Jesús Mingorance[1,6]

[1]Clinical Microbiology and Parasitology Department, Hospital Universitario La Paz, IdiPAZ, Madrid, Spain.
[2]Pediatric Intensive Care Unit, Hospital Universitario La Paz, Madrid, Spain.
[3]Preventive Medicine Department, Hospital Universitario La Paz, Madrid, Spain.
[4]Department of Pharmacy, Hospital Universitario La Paz, Madrid, Spain.
[5]Pediatric Tropical and Infectious Diseases Department, Hospital Universitario La Paz, Madrid, Spain.
[f]CIBERINFEC, Instituto de Salud Carlos III, Madrid, Spain

***Correspondence:**
Elias Dahdouh; Clinical Microbiology and Parasitology Department, Hospital Universitario La Paz, IdiPAZ, Paseo de la Castellana, 261, 28046, Madrid, Spain; elie.dahdouh@gmail.com

Supplementary Table 1. Sensitivity and specificity of the antibiotic-selective cultures used in this study compared to the PCR results for OXA-48 and VIM-producing organisms. Numbers in parentheses () indicate the range of the 95% Confidence Interval.

	Antibiotic-selective Cultures			
**PCR**	**OXA-48**		**VIM**	
	**Positive**	**Negative**	**Positive**	**Negative**
**Positive**	34	32	48	78
**Negative**	5	306	5	243
**Sensitivity**	51.52% (38.88 – 64.01)		38.1% (29.59 – 47.17)	
**Specificity**	98.39% (96.29 – 99.48)		98.98% (95.36 – 99.34)	

Supplementary Table 2. Observed genera, α-diversity (Shannon method), and percent relative loads (%RLs) of the four genes tested for in this study for the 40 samples selected for *16sDNA* metagenomic sequencing. "-" implies that the sample was negative for the corresponding gene.

Sample	Observed Genera	α-Diversity	%RL *bla*$_{CTX-M-1-Family}$	%RL *bla*$_{OXA1}$	%RL *bla*$_{OXA48}$	%RL *bla*$_{VIM}$
**S1**	11	0.595	-	-	-	21.58
**S2**	6	0.796	3.88	3.64	5.46	19.31
**S3**	15	2.055	4.21	4.81	4.80	13.09
**S4**	11	1.098	7.68	4.26	9.36	0.98
**S5**	17	0.856	6.04	5.78	8.56	1.66
**S6**	11	1.190	6.19	17.62	7.91	6.34
**S7**	32	2.292	0.00	0.00	0.00	8.29
**S8**	11	1.115	1.33	1.69	9.54	-
**S9**	11	1.420	2.67	3.17	6.15	-
**S10**	7	1.147	4.16	5.10	-	0.51
**S11**	14	1.764	2.20	2.35	2.41	0.05
**S12**	49	2.207	1.04	0.77	1.61	2.90
**S13**	6	1.165	1.17	1.51	2.94	-
**S14**	11	1.084	1.65	1.41	3.30	-
**S15**	13	1.365	2.49	4.06	0.00	-
**S16**	6	0.952	0.92	1.30	2.16	-
**S17**	15	1.654	0.66	0.61	1.52	-
**S18**	8	1.598	1.14	1.62	-	0.01
**S19**	9	0.325	0.14	0.18	0.55	0.94
**S20**	5	0.872	0.16	0.24	0.54	-
**S21**	23	1.712	0.29	0.37	-	-
**S22**	17	1.691	0.04	0.06	0.05	-
**S23**	12	1.524	0.00	0.00	0.00	-
**S24**	40	2.231	0.05	0.09	0.10	-
**S25**	7	0.489	0.15	0.15	0.16	-
**S26**	10	1.282	0.03	0.03	0.09	-
**S27**	12	0.713	0.03	0.03	0.09	-
**S28**	13	1.327	0.001	0.002	0.001	-
**S29**	3	0.788	0.001	-	0.003	0.001
**S30**	17	1.336	-	-	-	-
**S31**	16	1.882	-	-	-	-
**S32**	30	2.326	-	-	-	-
**S33**	37	2.259	-	-	-	-
**S34**	33	1.537	-	-	-	-
**S35**	34	2.010	-	-	-	-
**S36**	19	1.455	-	-	-	-
**S37**	18	2.139	-	-	-	-
**S38**	43	0.133	-	-	-	-
**S39**	28	0.409	-	-	-	-
**S40**	3	0.695	-	-	-	-
